# Counting Cats: Spatially Explicit Population Estimates of Cheetah (*Acinonyx jubatus*) Using Unstructured Sampling Data

**DOI:** 10.1371/journal.pone.0153875

**Published:** 2016-05-02

**Authors:** Femke Broekhuis, Arjun M. Gopalaswamy

**Affiliations:** 1 Mara Cheetah Project, Kenya Wildlife Trust, Nairobi, Kenya; 2 Wildlife Conservation Research Unit, Department of Zoology, University of Oxford, Recanati-Kaplan Centre, Tubney, United Kingdom; 3 Department of Zoology, University of Oxford, Oxford, United Kingdom; 4 Statistics and Mathematics Unit, Indian Statistical Institute–Bangalore centre, Bengaluru, India; Université de Sherbrooke, CANADA

## Abstract

Many ecological theories and species conservation programmes rely on accurate estimates of population density. Accurate density estimation, especially for species facing rapid declines, requires the application of rigorous field and analytical methods. However, obtaining accurate density estimates of carnivores can be challenging as carnivores naturally exist at relatively low densities and are often elusive and wide-ranging. In this study, we employ an unstructured spatial sampling field design along with a Bayesian sex-specific spatially explicit capture-recapture (SECR) analysis, to provide the first rigorous population density estimates of cheetahs (*Acinonyx jubatus*) in the Maasai Mara, Kenya. We estimate adult cheetah density to be between 1.28 ± 0.315 and 1.34 ± 0.337 individuals/100km^2^ across four candidate models specified in our analysis. Our spatially explicit approach revealed ‘hotspots’ of cheetah density, highlighting that cheetah are distributed heterogeneously across the landscape. The SECR models incorporated a movement range parameter which indicated that male cheetah moved four times as much as females, possibly because female movement was restricted by their reproductive status and/or the spatial distribution of prey. We show that SECR can be used for spatially unstructured data to successfully characterise the spatial distribution of a low density species and also estimate population density when sample size is small. Our sampling and modelling framework will help determine spatial and temporal variation in cheetah densities, providing a foundation for their conservation and management. Based on our results we encourage other researchers to adopt a similar approach in estimating densities of individually recognisable species.

## Introduction

Obtaining accurate estimates of densities is central to our understanding of the spatio-temporal dynamics of animal populations and provides the foundation for effective wildlife management and conservation. This is especially the case for large carnivores as many species have undergone drastic declines over the last few decades [1]. However, obtaining accurate density estimates of carnivores can be challenging as carnivores naturally exist at relatively low densities and are often elusive and wide-ranging [2]. As a result, carnivore populations are not always monitored using rigorous methods. For example, indirect methods such as spoor surveys are commonly used to estimate densities [3, 4]. To minimise cost and resources, such surveys are commonly conducted within a relatively small area and then extrapolated across a much larger area using index-calibration methods. However, this approach is inaccurate as the variations of detection probability are left unaccounted for [5]. As a result, population estimates could be over- or underestimated, misdirecting conservation efforts. In light of the consequences of these inaccuracies it is being recognised that more robust ways of estimating population parameters are required [6].

Over the last couple of decades, photographic capture-recapture methods [7] have been widely used in camera-trapping studies to estimate densities of various large felids, including tigers (*Panthera tigris* [8]), snow leopards (*P*. *uncia* [9]) and jaguars (*P*. *onca* [10]). In recent times, basic capture-recapture methods [11] have been replaced by spatially explicit capture-recapture methods (SECR [12, 13]) for estimating animal densities of `marked’ animals, wherein, spatial locations of each ‘capture’ are explicitly accounted for in the modelling. This has been a major advancement as not only can densities be accurately estimated across an entire landscape, but within-patch variation in densities can be estimated and can be suitably modelled with covariates of interest (e.g. [9]). The power of the SECR approach has led to an explosive growth of spatial capture-recapture methodologies (see [14] for a variety of models and potential variants). For example, SECR models were traditionally the domain of structured study designs such as camera trap arrays but are now adapted to unstructured sampling designs such as search-encounter data [15, 16]. This is an important development since although structured camera-trap study designs work well for species in forests and dense habitats, where animals generally use well-defined trails, they are not necessarily suited to species such as the cheetah (*Acinonyx jubatus*) that reside in open habitats where camera-trapping is likely to yield very low trapping rates leading to poor inferences [17].

It has been guesstimated that there are approximately 6,600 adult cheetah left on the African continent and that this number is continuing to decline [18]. Extinct in 20 countries and occupying only 17% of their historic range, cheetahs are vulnerable to extinction and in serious need of conservation efforts [1]. Whilst accurate density estimates are crucial for conservation efforts to be successful, these are not always available. For example, Kenya is considered to be a critical part of the global cheetah distribution but there are currently no accurate cheetah population density estimates in any area in the country [19, 20]. Within Kenya, the Maasai Mara, which is part of a larger Mara-Serengeti transboundary region, is believed to hold one of the country’s main cheetah populations. The Maasai Mara is renowned for its annual migration of wildebeest (*Connochaetes taurinus*) and high densities of predators but, like many landscapes around the world, it is under increasing anthropogenic pressure. Kenya’s population is now over 41.8 million, over three times what it was in 1970 [21]. In the Maasai Mara, settlements, and therefore also livestock, are increasing at a rapid rate [22]. As a result, this area has seen significant declines in herbivore numbers, some species down by a third, in the last few decades [23]. While there are concerns that the cheetah population in the Maasai Mara is facing similar declines, there are no accurate population estimates to support this or to determine future population trends. In this study, we aim to provide the first rigorous estimate of the cheetah population in the Maasai Mara National Reserve and the surrounding wildlife conservancies using a sex-specific SECR modelling approach based on field sampling via direct sightings of individual cheetah using an unstructured spatial sampling design.

## Methods

### Ethics Statement

Data for this study were collected using non-invasive methods and therefore approval from an ethics committee was not required. Permits for this study were issued to Femke Broekhuis by the National Council for Science and Technology (NACOSTI), Kenya Wildlife Service (KWS), Narok County Government (NCG), the Maasai Mara Wildlife Conservancies Association (MMWCA) and the Mara Conservancy.

### Study area

**Fig 1:**
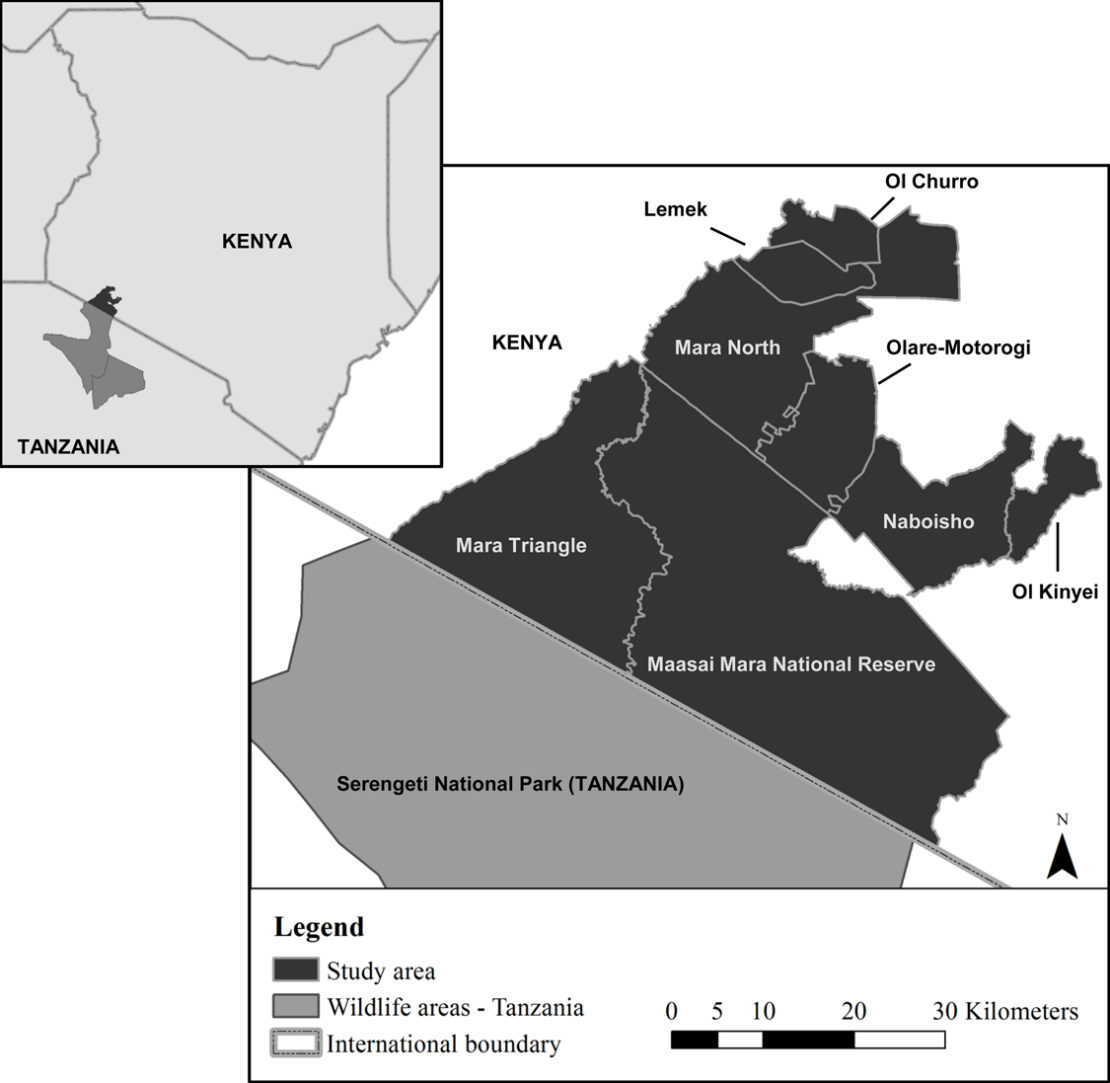
Map of the study area in South-west Kenya (dark grey) bordering Serengeti National Park in Tanzania (light grey).

The survey was conducted in the Maasai Mara located in the South-west of Kenya. The study area (centred at °1 S, 35°E; elevation c. 1700 m) covers approximately 2398km^2^ which includes the Maasai Mara National Reserve (MMNR), which falls under the authority of the Narok County Government, and the adjacent conservancies; Mara Triangle, Mara North, Ol Churro, Lemek, Olare-Motorogi, Naboisho and Ol Kinyei which are managed by private management companies (Fig 1). There are no physical barriers between the MMNR and the conservancies or between the wildlife areas and the surrounding community areas, allowing for free movement of animals. Hereafter, the MMNR and the adjacent conservancies will collectively be referred to as the Maasai Mara. To the south the Maasai Mara borders the Serengeti National Park in Tanzania, to the north and west it borders intensive agricultural land and east of the Maasai Mara is largely pastoralist settlement [24, 25].

The study area experiences a bimodal rainfall pattern, with the wet season spanning November–June and the dry season July–October. The wet season is characterised by two distinct periods; the short rains (November–December) and the long rains (March to June) [26]. The long rains attract the migrating wildebeest, Common zebra (*Equus burchelli*) and Thomson’s gazelle (*Gazella thomsoni*) from the Serengeti. Generally the migration reaches the Northern Serengeti in July, and then spends August, September and October in the Maasai Mara before returning to Tanzania in November [27, 28]. Independent of the migration, substantial populations of Thomson’s gazelle—cheetahs preferred prey in East Africa [29]—are resident year round as are other prey species such as Grant’s gazelle (*G*. *granti*), impala (*Aepyceros melampus*) and hares (*Lepus spp*.*)* [28, 30].

### Field methods

**Fig 2:**
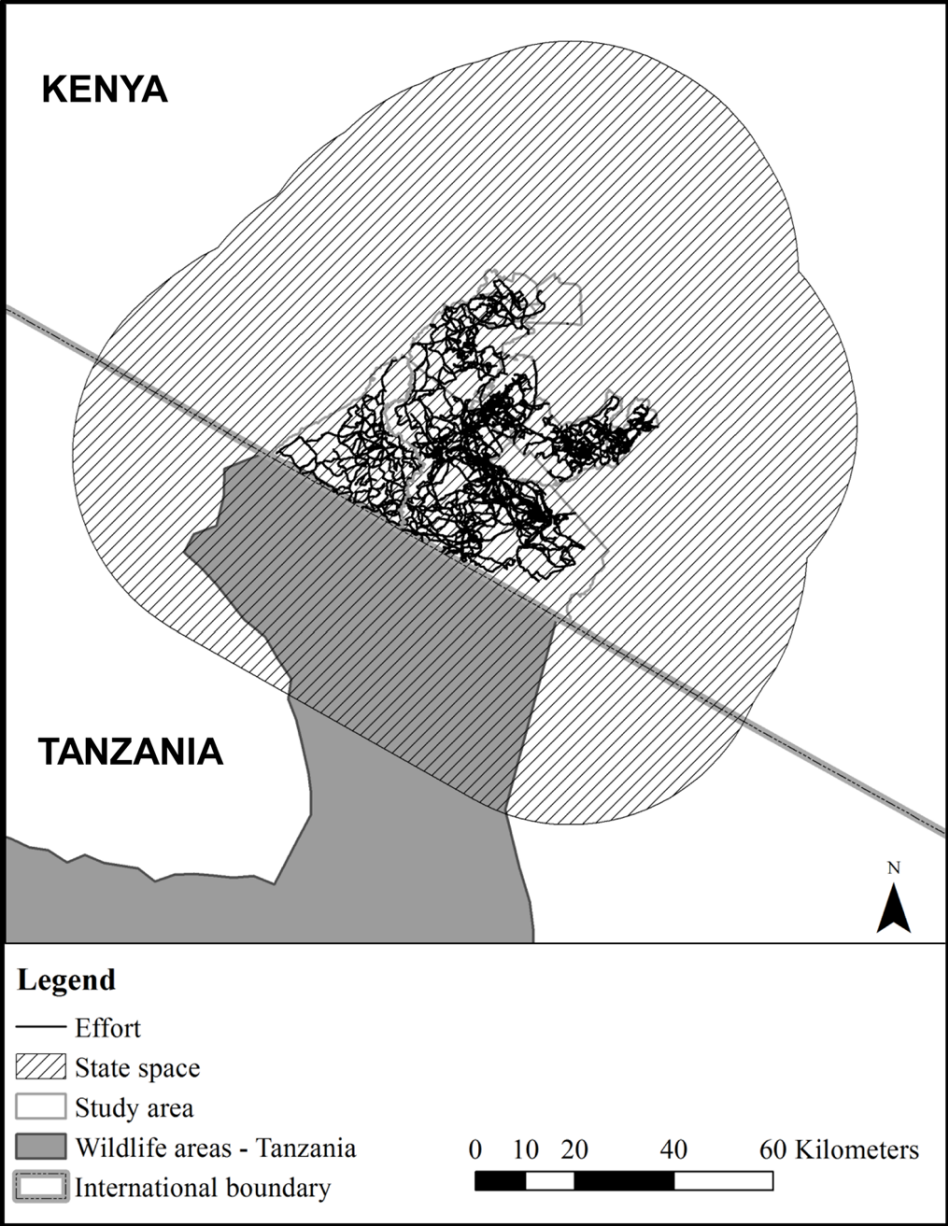
Map of the Maasai Mara including the sampling effort (tracks driven) in search of cheetah and the state space.

An intensive cheetah survey was conducted during a three-month period from 1^st^ August 2014 to 31^st^ October 2014. Based on other studies, we believe that this was short enough so not to seriously violate the assumption of closure but long enough to collect sufficient data [9, 17, 31]. The survey involved a maximum of five vehicles traveling within the study area with the objective of adequately covering the entire study area spatially. Whenever cheetahs were sighted, photographs of each individual were taken and the number of individuals, GPS location, date and time of day were recorded. In addition to recording opportunistic sightings of cheetahs during sampling, we also made use of information on cheetah locations provided by tourists, guides and rangers, so that our field teams would drive to the location and photo-capture cheetahs. Sampling effort (GPS tracks) and cheetah sightings were recorded using an application built in Cybertracker v3 [32]. To account for search effort, GPS locations were recorded every 10 seconds giving a detailed record of the areas that were covered in search of cheetahs (Fig 2). Whenever possible, routes travelled were not used more than once a day to ensure that cheetahs were not sighted multiple times per sampling occasion. Each cheetah was identified according to their unique spot pattern [33]. Only independent, adult cheetah were used for the analysis and in the case of male coalitions each individual was considered to be independent in the analysis.

### Analytical methods

Cheetah densities in the Maasai Mara were estimated using a Bayesian spatially explicit capture-recapture model adapted to search-encounter data and sex differences in encounter probability [34]. The definitions of the parameters used for the analyses can be found in Table 1 and details on the input variables are described in more detail:

**Table 1:** Parameters used in the sex specific spatially explicit capture-recapture analysis for cheetah in the Maasai Mara, Kenya

Parameter	Definition
*n*	Total number of cheetah sighted during the sampling period.
*n*_*z*_	Number of cheetah augmented to *n*, so that *M* = *n*+*n*_*z*_ represents the maximum number of cheetahs in the large state space *S*.
*σ*_*F*_	Rate of decline in detection probability as a female cheetah’s activity centre increases as a function of her distance from the centroid of a grid cell (or trap).
*σ*_*M*_	Rate of decline in detection probability as a male cheetah's activity centre increases as a function of his distance from the centroid of a grid cell (or trap).
β_*sex*_	Difference in the complementary log-log value of detection probability between a male and female cheetah.
β_*eff*_	Rate of change in the complementary log-log value of detection probability, as the log(effort) changes by one unit. Here, unit of effort is one kilometre driven.
*λ*_*0*_	Basal encounter rate of a cheetah whose activity centre is located exactly at the centroid of a grid cell.
*ψ*	Ratio of the true number of individuals in the population compared to the data augmented population *M*.
*ψ*_*sex*_	Proportion of cheetah that are male. Sex ratio = $\frac{1-\psi sex}{\psi sex}$ females:males
*N*_*super*_	Total number of cheetah in the larger state space *S*.
*ϑ*	Determines the shape of the estimated detection function. The value of *ϑ* ranges from 0.5 (Exponential) to 1 (Gaussian).
*D*	Estimated density of adult cheetah/100km^2^

#### State space

The large statespace *S* was defined as the study area with a buffer of 40km resulting in an area of 20,370km^2^ (Fig 2). All habitats were considered suitable apart from intense agricultural areas towards the north and west of the study area and large, densely populated towns [24]. Unsuitable habitat accounted for 42.58% (8673.54km^2^) within the statespace which was masked out from the analysis [13, 35]. The statespace was represented in discrete 650 x 650m (0.422km^2^) pixels.

We assumed that, during the three-month sampling period, no cheetah outside this large statespace would be detected within the sampling area. While the maximum distance that was covered by a two-male coalition was 41km we believe this to be a reasonable assumption as the calculated Mean Maximum Distance Moved during this period for all cheetah sighted was 6.91km. This was further supported by the fact that photographs received of cheetahs in nearby areas, but outside this buffer, were not of individuals that were seen within the defined sampling area over a two year period. Within the large statespace the data-augmented value of cheetah abundance *M* was set at 335 (*n* = 25 and *n*_*z*_ = 310; see Table 1 for definitions of parameters). This represents the maximum number of cheetahs possible within the statespace, keeping in mind that our expectation for an estimate of the true number of individuals in the population compared to the data-augmented population *M*, *ψ*, will lie within the range 0.2–0.8 for reliable estimation of the binomial proportion [36].

$$\left[N_{super}|M,\psi ]∼\mathrm{Bin}[M,\psi \right]$$

#### Observation process

The data collected were summarised using a standard spatial capture-recapture 3-dimensional matrix (see [35] for a full example) that consists of individuals (i = 1; 2; 3: : : N), x trap locations (j = 1; 2; 3: : : J) and x sampling occasions (k = 1; 2; 3: : : K). This is a standard matrix used for spatial capture-recapture data for camera trap surveys which can be adjusted for unstructured spatial capture-recapture sampling [15, 34]. In this case, the trap locations were defined by 1km^2^ pixels to represent an array of ‘traps’. The sampling occasion, or ‘trap activity’, was based on whether a given pixel was surveyed on any particular day. However, since investing more effort in certain pixels over the others could yield a higher number of detections in highly sampled pixels, we incorporated an additional covariate of search effort (logarithm of the distance travelled in kilometres) per pixel per sampling occasion. In addition, we incorporated sex-specific covariates (female = 0 and male = 1) as it has been shown that sex differences in spatial distribution are likely to influence the observation process in spatial capture-recapture models [10]. This is especially true for carnivores where males and females tend to have different home range sizes [37, 38].

Spatial capture-recapture models employ a variety of detection function models [16, 39] to define the mechanism of the decline in detection probability as the distance between the activity centre of an animal from a trap location (in our case, the centroid of a pixel) increases. Rather than explicitly testing various detection function models, we consider an infinite number of possibilities between a perfect negative exponential detection function to a perfect Gaussian detection function [13] by explicitly estimating the parameter *ϑ*. A value of *ϑ* = 0.5 specifies a perfect negative exponential function which would indicate that an animal’s activity is concentrated around its activity centre which is indicative of increased fidelity to the activity centre. A value of *ϑ* = 1 specifies a perfect Gaussian function which would indicate that an animal’s activity is more widespread and less concentrated in a particular area which may be characteristic of animals that have home-ranges with a lesser degree of fidelity to the activity centre. Thus, the parameter *ϑ* defines the shape of the detection function, and is indicative of the resource utilisation mechanism of cheetahs in our example. Hence, the probability of detecting a cheetah *i*, in sampling occasion *k* at pixel *j*, *π*_*ijk*_, is defined by a complementary log-log function of covariates. Based on this, the full model is: cloglog (*π_ijk_*) = logλ_0_ + β_*eff*_[log(EFFORT_*jk*_)] + β_*sex*_ (SEX) - *f*[dist(i,j|*ϑ,σ_sex_*)]

#### Bayesian models

The following four *a priori* models were defined:

Model 1: Full model that assumes detection probability is sex-specific and the detection function shape is estimated (defined by *ϑ*).

$$\left[\beta_{sex},\vartheta (.)\right]$$

Model 2: Assumes detection probability is not a function of sex. However, rate of decline in detection probability *σ* remains sex-specific, since this parameter is also related to animal movement.

$$\left[\beta_{sex}=0\left(\mathrm{fixed}\right),\vartheta (.)\right]$$

Model 3: Detection probability does not vary between sex and detection function shape, defined by *ϑ*, is fixed at a certain value (implying a fixed hybrid model between a Gaussian and a negative exponential detection function).

$$\left[\beta_{sex}=0\left(\mathrm{fixed}\right),\vartheta =0.75(\mathrm{fixed})\right]$$

Model 4: Detection probability is sex-specific but the shape of the detection function is fixed.

$$\left[\beta_{sex},\vartheta =0.75\right]$$

These four *a priori* Bayesian SECR models were implemented using an adaptation of the package SCRbayes (https://github.com/jaroyle/SCRbayes) in the programming environment R [40]. The models were implemented using the Bayesian Markov Chain Monte Carle (MCMC) simulation using the Metropolis-Hastings algorithm [41]. The MCMC chain convergence for all the models was assessed using the Gelman-Rubin diagnostic [42]. Each model was set for 11,000 iterations with a burn-in period of 1,000 iterations. The burn-in period was refined during later stages of an analysis if there was evidence from the diagnostics that the model had not arrived at a stationary distribution by then. As a result there were 4000–5000 posterior samples for each chain. A total of eight chains were run for each model. For each model, a Beta(1,1) prior was used for *ψ*. This prior is intended to be uninformative, which is why we made sure that *M* was large enough so that the estimate of *ψ* was much less than 1. We therefore believe that the problem of a potentially truncated posterior, as observed in Link [43], does not apply in our case. In addition, all the coefficients in the linear predictor had improper flat priors on [- ∞; ∞] for a suitable transformation of the parameter. The intercept was *ln*(λ_0_), where λ_0_ was the expected number of captures of a single animal in a single trap on a single capture occasion, when the distance between the trap and the centre of the animal's home range is zero. This translated, therefore, into implied scale priors for *λ*_*0*_ and *β*_*effort*_. The *ϑ* had a *Uniform*(0.5, 1) prior to facilitate the shape of the resource selection function to have an equal probability to be any form between a half-normal and a negative exponential detection function. A flat *Uniform*[0, ∞] prior was used for σ and activity centres had uniform priors, so that all pixels (potential home-range centres) with suitable habitat had an equal probability of hosting a home-range centre whereas pixels with unsuitable habitat had zero probability of hosting a home-range centre. Each model was subsequently checked for adequacy utilising the Bayesian p-value assessment using a test statistic based on individual encounters as implemented in Royle et al. [13]. Parameter estimates are reported together with the posterior standard deviations.

## Results

The results of the four models predict that the density for cheetahs in suitable habitat within the Maasai Mara landscape lies between 1.28 ± 0.315 and 1.34 ± 0.337 adults/100km^2^. These results are based on 59 sightings of 25 individuals (nine males were sighted on 25 occasions and 16 females were sighted on 34 occasions) that were sighted over an invested sampling effort of 8397km during a three-month period. The number of sightings per individual ranged from one to seven and the basal encounter rate (*λ*_*0*_), implying that the probability of sighting a cheetah per kilometre driven = 1-exp(-*λ*_*0*_), was 0.004.

**Fig 3:**
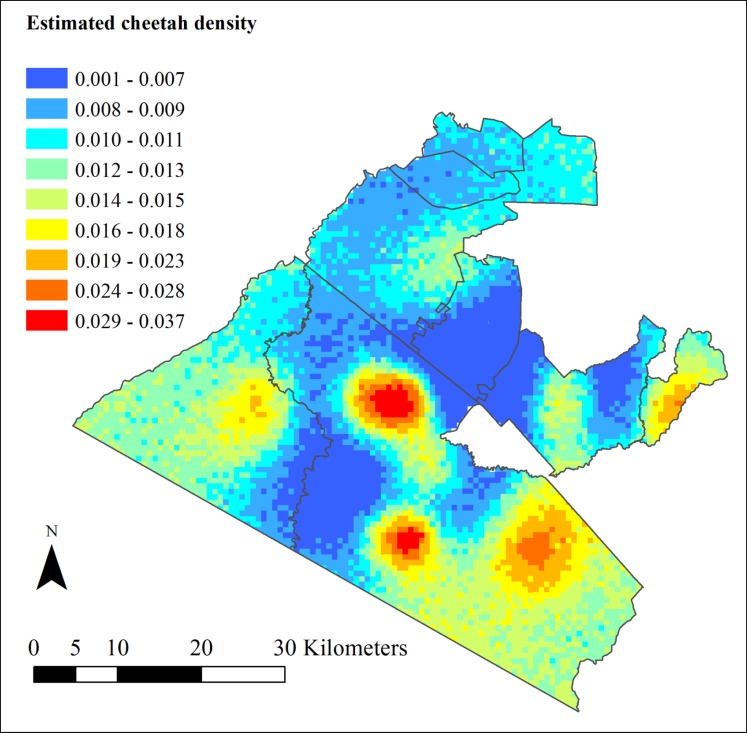
Map of the estimated posterior density of cheetah in the Maasai Mara, Kenya for each 0.422km^2^ pixel for the period between 1^st^ August and 31^st^ October 2014. The cheetah density/km^2^ per pixel ranged from 0.001 (dark blue) to 0.037 (red).

The analysis provides estimates for various other parameters, including a sex ratio of approximately five females to every one male. There was however a high sampling covariance between density (*D*) and the sex ratio (*ψ*_*sex*_) as evident from pairwise covariance plots of parameters from the MCMC output. The estimated detection function (*ϑ*) was closer to 1 than to 0.5 indicating that the shape was closer to Gaussian rather than a negative exponential. Fig 3 depicts the expected pixel-specific posterior densities and shows the heterogeneous distribution of cheetah within the landscape. The expected number of cheetah/ km^2^ per pixel ranged from 0.001 to 0.037 showing clear ‘hotspot’ areas for cheetah.

The posterior mean estimates along with posterior standard deviations of these estimates of the parameters for Models 1–4 and the Gelman-Rubin statistic are reported in Table 2. The Bayesian p-value estimates for all the models are between 0.67–0.69 indicating that all the models were adequate, as the values lie well within the extremities (0.15–0.85).

**Table 2:** Posterior estimates of parameters for Models 1–4 including the posterior standard deviation (PSD). Density (*D*) is given as number of adult cheetah/100km^2^

Model	Model 1 [β_*sex*_, θ(.)]		Model 2 [β_*sex*_ = 0(fixed), θ(.)]		Model 3 [β_*sex*_ = 0(fixed), θ = 0.75 (fixed)]		Model 4 [β_*sex*_, θ = 0.75 (fixed)]	
**Number of posterior samples used**	**4000**		**5000**		**5000**		**5000**	
**Maximum value of potential reduction factor**	**1.18**		**1.12**		**1.11**		**1.13**	
**Bayesian P-value**	**0.6729**		**0.6838**		**0.6767**		**0.6732**	
**Parameter**	**Posterior mean**	**PSD**	**Posterior mean**	**PSD**	**Posterior mean**	**PSD**	**Posterior mean**	**PSD**
***σ***_***F***_	3.660	1.272	3.300	1.241	2.740	0.331	2.690	0.303
***σ***_***M***_	8.130	3.589	7.460	3.814	5.330	0.748	5.810	1.337
**β**_***sex***_	-0.120	0.531	-	-	-	-	-0.140	0.596
**β**_***eff***_	-0.009	0.012	-0.009	0.012	-0.009	0.012	-0.009	0.013
***λ***_***0***_	0.004	0.002	0.004	0.002	0.004	0.001	0.004	0.002
***ψ***	0.450	0.112	0.460	0.116	0.450	0.111	0.469	0.120
***ψ***_***sex***_	0.162	0.073	0.146	0.068	0.159	0.071	0.146	0.700
***N***_***super***_	149.800	36.780	153.100	37.960	151.14	36.352	157.160	39.390
***ϑ***	0.831	0.127	0.792	0.134	-	-	-	-
***D***	1.28	0.315	1.33	0.326	1.29	0.311	1.34	0.337

## Discussion

The results indicate that sex-specific SECR models for unstructured spatial sampling can be used successfully to estimate population parameters for low density species that reside in open habitats. This is especially the case when covering a large area in a short period of time so as to minimise violating the assumption of a closed system. While the posterior variances of the expected density estimates are relatively high, this is an inherent problem when sample sizes are small. In the past, cheetah densities have been estimated using both indirect survey methods such as interviews [44] and spoor surveys [3] but these techniques have their limitations. With both methods there is the uncertainty associated with the misidentification of cheetah and their spoor, especially as they are often confused with leopard (*P*. *pardus*) and therefore estimates based on indirect methods might be misrepresentative. Even without misidentification errors these approaches can be highly inaccurate, especially when detection probabilities are low and varying due to a high amount of overdispersion [5]. As a result, changes in population estimates cannot be accurately estimated, consequently misdirecting conservation decisions. In light of these inaccuracies it is increasingly recognised that ecologists must embrace universally robust methods that explicitly recognise the importance of estimating detection probabilities [6].

Capture-recapture models that take into account detection probability, have been used in camera-trap studies to estimate cheetah densities in Algeria [45], Namibia [46], South Africa [47] and Botswana [17, 48]. Cheetah, however, occur at low densities and traverse large areas so in areas with open habitats, camera trapping methods are likely to yield very low trapping rates and the resulting estimates of parameters are likely to have high sampling variances and covariances. Furthermore, while camera-trap studies are feasible for small areas like those in Botswana (240km^2^ [17]), Namibia (277km^2^ [46]) and South Africa (300km^2^ [47]), larger areas are logistically difficult to maintain, costly and, if the adjacent block method is used, are likely to be confounded by spatial and temporal variation [49]. In addition, the mentioned studies used basic capture-recapture models that do not account for variable trap effort (inevitable in field sampling) or for the spatial arrangement of individuals [13], which often leads to an upwards bias in density estimates (see [9, 14]).

Generally in large carnivores, males and females differ in ranging behaviour and hence detection rate is likely to differ between sexes [37]. In such circumstances estimates can be improved by accounting for sex [10]. Moreover, by including sex as a parameter, the sex ratio and sex-specific movement parameters of the population can be determined. For cheetah the sex ratio at birth tends to be equal [50] but adult females have a higher chance of survival than males and in the Serengeti National Park in Tanzania the adult sex ratio was found to be 0.8 males for every female [51, 52]. We would therefore expect a similar sex ratio in the Maasai Mara but instead the sex ratio was highly skewed towards females with about one male for every five females. However, we observed a high negative sampling covariance between density (*D*) and the sex ratio (*ψ*_*sex*_). This covariance is indicative of some parameter redundancy between *D* and *ψ*_*sex*_ implying that this cheetah dataset may be insufficient to distinguish between these two parameters. The solution to such a problem, if obtaining larger sample sizes is not a realistic option, is to provide informative priors for one of the parameters, say *ψ*_*sex*_, and re-run the analysis. But in order to do this, we must be certain that the prior information comes from a reliable source. So far, estimates of sex ratios in cheetah populations only come from ‘observed’ individuals, and if there is an underlying problem of detection biases associated with these observations, then the estimated sex ratios will also be biased. This is not an unrealistic situation to expect, because in many populations transients tend to have lower detection probabilities [53]. Hence, we advise against using such informative priors that may contain inherent detection biases. That said, in this specific cheetah analysis, we suspect that *ψ*_sex_ has been estimated to be very low, implying an upward bias in the estimates of density. If this is the case then this would further strengthen our believe that estimated cheetah densities are lower than previously thought [44]. On the other hand, if the sex ratio estimated is accurate then this raises concerns as it would suggest that male survival, based on figures from the Serengeti, is drastically lower than expected.

Previous studies have found that non-territorial individuals (females and semi-nomadic males) have larger home-ranges than territorial males [50, 54]. While this might be true over a large time-scale, it might not be the case for a small timeframe of three months. Firstly, five of the 16 females that were sighted had cubs, including three with cubs younger than four months, which would have severely restricted their movement [55, 56]. Secondly, male cheetah in this study had a Mean Maximum Distance Moved (MMDM) of 9.49km which was almost twice the MMDM of female cheetah of 5.46km. Males are generally more active than females [57], possibly because they need to patrol their territory [58]. It is therefore likely that, over a short timeframe, males cover more distance than females. Lastly, the movement of female cheetah is primarily governed by the movement of their preferred prey, Thomson’s gazelle [59]. Territorial males on the other hand tend not to be influenced by the movement of prey unless food becomes particularly scarce [58]. As the survey was conducted during the annual migration when Thomson’s gazelle are in abundance in the Maasai Mara, it is possible that females were concentrated in prey-rich areas [28].

The expected cheetah density in the Maasai Mara, which lies between 1.28 ± 0.315 and 1.34 ± 0.337 adult cheetahs/100km^2^, is higher than the estimated densities of cheetah in other areas in Africa such as Algeria (0.02–0.05/100km^2^ [45]) and Botswana (0.61 ± 0.18 cheetahs/100km^2^ [17]). As the Maasai Mara is part of a much larger Mara-Serengeti ecosystem we expected our density estimates to be similar to those in the Serengeti. While our estimated cheetah density in the Mara is indeed similar to the 0.8–1.0 adults/100km^2^ estimated by Caro [50] it is lower than a more recent estimate by Durant et al. [60] of 2 individuals/100km^2^. It is possible that the density of cheetah in the Maasai Mara is lower than the Serengeti as there is a larger edge effect present in the Maasai Mara, thereby generating a possible sink for cheetahs in this landscape [61]. However, it is important to note that it is difficult to compare the density obtained in this study to the densities obtained in the Serengeti as different methodologies were used: Caro [50] used data from a whole count (which assumes that every individual is counted) whereas Durant et al. [60] used a distance based method (which may be inefficient in the context of losing information on individual identities). To fully understand cheetah density and variations across the Mara-Serengeti landscape and other areas it would be beneficial if similar, and robust methodologies [6] are adopted throughout the cheetah range.

The SECR models used in this study not only provide a general population estimate, they also provide the spatial variation in densities (Fig 2). Generally carnivore densities are correlated to prey biomass [62]. However for mesopredators, like cheetah, this may not always be the case as populations can be suppressed by top-down effects such as intraguild predation and competition ([63, 64] but see [65]). It is believed that cheetah avoid lion (*P*. *leo*) because they have a negative impact on cheetah as they kill both cubs and adults and steal their kills [66, 67]. However, Broekhuis et al. [68] showed that, in the long-term, cheetah use similar habitats and areas to lion which is possibly related to the availability of prey. This is supported by findings of Durant et al. [59] who found that cheetah distribution and movement are positively correlated to the presence of prey [59]. On an individual level the spatial distribution of some mesopredators is a hierarchical process, first driven by resource acquisition and thereafter fine-tuned by predator avoidance [68]. It is however unclear whether the same applies at a population level i.e. areas where multiple individuals tend to congregate or avoid. It is possible that environmental factors, such as habitat, prey, predators and anthropogenic factors such as livestock grazing, influence the spatial variation of carnivore densities [9]. The results presented here provide the spatially explicit data needed to further investigate both the environmental and anthropogenic variables that determine the spatial variation in carnivore densities. Understanding variations in densities over both space and time is key for the conservation and management not just of single species, but of communities and landscapes.
